# Fourier transform infrared spectroscopy for *Streptococcus pneumoniae* capsular serotype classification in pediatric patients with invasive infections

**DOI:** 10.3389/fmicb.2024.1497377

**Published:** 2024-11-21

**Authors:** Thaís Muniz Vasconcelos, Luiza Souza Rodrigues, Damaris Krul, Sabrina da Conceição Barbosa, Adriele Celine Siqueira, Samanta Cristine Grassi Almeida, Ana Paula de Oliveira Pacheco Souza, Marcelo Pillonetto, Rodrigo Oliveira, Carolyn Gertruda Josephina Moonen, Christian de Alencar Siebra, Libera Maria Dalla-Costa

**Affiliations:** ^1^Faculdades Pequeno Príncipe, Curitiba, Paraná, Brazil; ^2^Instituto de Pesquisa Pelé Pequeno Príncipe, Curitiba, Paraná, Brazil; ^3^Laboratório Central do Estado do Paraná (LACEN/PR), São José dos Pinhais, Brazil; ^4^Instituto Adolfo Lutz (IAL), São Paulo, Brazil; ^5^Hospital Pequeno Príncipe (HPP), Curitiba, Paraná, Brazil; ^6^Bruker Daltonics GmbH & Co. KG, Bremen, Germany

**Keywords:** *Streptococcus pneumoniae*, FT-IR spectroscopy, invasive pneumococcal disease, serotypes, pediatrics

## Abstract

Invasive pneumococcal disease (IPD) is a major cause of morbidity and mortality worldwide, particularly in the pediatric population (children and infants), with high rates of hospitalization and death. This study aimed to create and validate a classifier for *Streptococcus pneumoniae* serotyping using Fourier-transform infrared (FT-IR) spectroscopy as a rapid alternative to the classical serotyping technique. In this study, a database comprising 76 clinical isolates, including 18 serotypes (predominantly serotypes 19A, 6C, and 3) of *S. pneumoniae* from pediatric patients with IPD, was tested at a tertiary pediatric hospital in southern Brazil during 2016–2023. All isolates were previously serotyped using the Quellung reaction, and 843 FT-IR spectra were obtained to create a classification model using artificial neural network (ANN) machine learning. After the creation of this classifier, internal validation was performed using 384 spectra as the training dataset and 459 as the testing dataset, resulting in a predictive accuracy of 98% for serotypes 19A, 6, 3, 14, 18C, 22F, 23A, 23B, 33F, 35B, and 9N. In this dataset, serotypes 10A/16F, 15ABC, and 7CF could not be differentiated and were, therefore, grouped as labels. FT-IR is a promising, rapid, and low-cost method for the phenotypic classification of *S. pneumoniae* capsular serotypes. This methodology has significant implications for clinical and epidemiological practice, improving patient management, monitoring infection trends, and developing new vaccines.

## 1 Introduction

*Streptococcus pneumoniae* colonizes the mucosal surfaces of the human upper respiratory tract, causing opportunistic, non-invasive, and invasive infections. Invasive pneumococcal disease (IPD) is a severe condition with high morbidity and mortality rates worldwide, notably affecting children under 5 years of age (Weiser et al., 2018). Vaccination is the most effective way to protect the general population from IPD (Lages et al., 2020). There are differences in the distribution of pneumococcal serotypes among patients with IPD worldwide (Silva-Costa et al., 2018). To date, 106 different capsular serotypes have been described and categorized (Ganaie et al., 2020; Ganaie et al., 2023b; Ganaie et al., 2021; Ganaie et al., 2023a; Manna et al., 2024). However, only a few pneumococcal serotypes cause the vast majority of IPD cases worldwide, and different vaccines have been developed to target these prevalent serotypes (Ganaie et al., 2020; Qian et al., 2021). The first vaccine developed to control pneumococcal infections, the 23-valent pneumococcal polysaccharide, was introduced in 1983. However, due to the increase in penicillin-resistant pneumococci and also the observation that non-conjugate vaccines produced unsatisfactory results in children under 2 years of age, the first pneumococcal conjugate vaccine (PCV), including seven pneumococcal serotypes (PCV7, serotypes 4, 6B, 9 V, 14, 18C, 19F, and 23F), was developed and authorized for use in children in the USA in 2000 (Rappuoli et al., 2019; Jarovsky and Berezin, 2023). Two new second-generation conjugate vaccines with additional serotypes, PCV10 (additional serotypes 1, 5, and 7F) and PCV13 (additional serotypes 3, 6A, and 19A), were licensed based on their non-inferior immunogenicity to PCV7 (Jarovsky and Berezin, 2023). Following the worldwide use of PCVs, the emergence of IPD due to non-vaccine serotypes and the development of new vaccines with a higher valency has emerged: PCV15, which adds serotypes 22F and 33F, has been approved for use in children and adults, and the PCV20 vaccine, which covers five more serotypes compared to PCV15 (8, 10A, 11A, 12F, and 15 B), was recently approved by the National Health Surveillance Agency (*Agência Nacional de Vigilância Sanitária* - ANVISA) (Jarovsky and Berezin, 2023; Kfouri et al., 2023; ANVISA AN de VS, 2023). The surveillance of circulating serotypes in the population is crucial for evaluating the impact of vaccination programs and understanding the distribution of circulating serotypes involved in IPDs (Bardach et al., 2024). In the literature, two main approaches to investigating pneumococcal capsular types are characterized, these being serological and molecular methods (Abdul Rahman et al., 2023). Molecular techniques include real-time PCR with amplification of the *lytA* gene, microarray using chips to detect and distinguish serotypes and whole genome sequencing (WGS) (Swarthout et al., 2021; Abdul Rahman et al., 2023; Donkor, 2013; Velusamy et al., 2020). The Quellung reaction, in which antisera are used and tested sequentially with pools of antisera until a positive reaction is observed in the identified pneumococcus strain, is considered the gold standard for identifying serotypes (Habib et al., 2014; Novais et al., 2019). The IR Biotyper^®^ system, a Fourier transform infrared spectroscopy (FT-IR)-based method, shows promise for capsular typing (Novais et al., 2019). IR spectroscopy provides a molecular fingerprint based on the absorption of infrared light (4,000–500 cm^−1^) by carbohydrates, lipids, proteins, and lipopolysaccharides (Muchaamba and Stephan, 2024). Specifically, the region of interest for capsular typing of *Streptococcus pneumoniae* focuses on carbohydrates, primarily characterized by absorbent properties (C-O stretching and O-H bending, 1,300–800 cm^−1^). This method has demonstrated potential for serotyping various bacteria. FT-IR has been previously reported in the literature as an effective serotyping technique for *S. pneumoniae*, with comprehensive validation of the method (Burckhardt et al., 2019; Passaris et al., 2022). This study aimed to create a database and validate a local classifier for pneumococcal serotyping using FT-IR spectroscopy, on isolates exclusively from pediatric patients, as a quick and easy-to-perform alternative to the Quellung reaction.

## 2 Materials and methods

### 2.1 Study design

**Figure 1:**
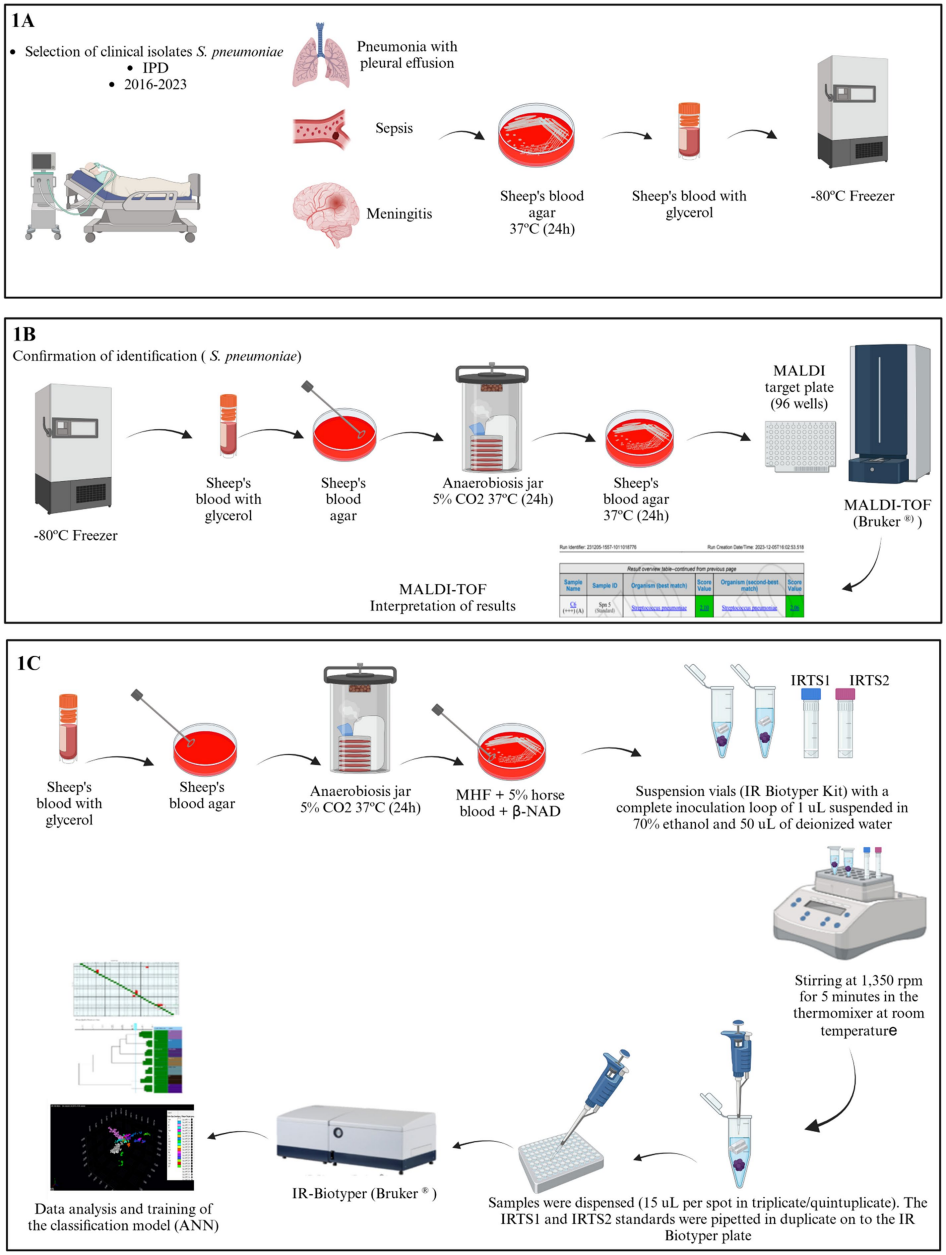
Overview of practical sequential workflows of this study. **(A)** Steps involved in the selection and cryopreservation of clinical isolates included in the study. **(B)** Species identification using MALDI-TOF for the study isolates. **(C)** Workflow of IR Biotyper analysis. This figure was created by Biorender.com.

This was a retrospective longitudinal study of 76 clinical isolates of *S. pneumoniae* from pediatric patients diagnosed with IPD admitted between 2016 and 2023 to Hospital Pequeno Príncipe, a 372-bed academic pediatric hospital in Curitiba, Paraná, southern Brazil. All isolates were stored in sheep blood with 20% glycerol at −80°C (Figure 1A).

### 2.2 Bacterial identification

Bacterial colonies were cultivated on 5% sheep blood Columbia agar base (CBAB 5%) (NEWPROV, Pinhais - PR, Brazil) at 37°C in an atmosphere of 5% CO_2_ for 24 h. Identification was carried out using matrix-assisted laser desorption ionization time-of-flight mass spectrometry (MALDI-TOF MS) (Bruker Daltonics GmbH, Bremen, Germany) on a Microflex LT Bruker MALDI-TOF MS with flexControl v3.4 software (Bruker Daltonics GmbH, Bremen, Germany) (Figure 1B). Confirmation was achieved by standard methods (WHO, 2011) species were identified by the susceptibility on the optochin test and on sodium deoxycholate (bile salt) solution.

### 2.3 *Streptococcus pneumoniae* serotyping

Serotyping was carried out at the Instituto Adolfo Lutz (IAL) of the National Reference Laboratory for Bacterial Meningitis and Invasive Pneumococcal Infections of the Brazilian Ministry of Health. *S. pneumoniae* was serotyped using pneumotest-latex agglutination and the Quellung reaction using antisera, both from Staten’s Serum Institute (Copenhagen, Denmark) and according to the manufacturer’s instructions. Non-typeable (NT) isolates identified by Quellung were also verified by the deduction of serotypes/serogroups using sequential multiplex PCR for 41 serospecificities, following the gene targets and protocols described by the Centers for Disease Control and Prevention, Atlanta, GA, United States (Carvalho et al., 2010).

### 2.4 Polysaccharide-based phenotypic typing with FT-IR spectroscopy

Polysaccharide-based phenotypic typing was conducted using the IR Biotyper^®^ system (Bruker Daltonics, Germany), located at Laboratório Central do Estado do Paraná (LACEN/PR). The selected bacterial isolates were first cultured on CBAB 5% (NEWPROV, Pinhais - PR, Brazil) at 37°C in 5% CO_2_ for 24 h. The obtained bacterial colonies were subsequently cultured on MHF agar (Mueller Hinton agar +5% horse blood +20 mg/L *β*-NAD, NEWPROV, Pinhais - PR, Brazil) and incubated at 37°C in 5% CO_2_. Suspensions of the bacterial isolates were prepared in IR Biotyper^®^ suspension vials containing metal beads (IR Biotyper^®^ kit, Bruker Daltonics GmbH, Bremen, Germany) according to the manufacturer’s instructions. Portions of the culture colonies were removed using a 1 μL disposable inoculation loop, and a homogeneous suspension was prepared in 50 μL of 70% ethanol. After homogenization, 50 μL of deionized water was added. The samples were then agitated at 1,350 rpm for 5 min (Thermomixer, Eppendorf, Hamburg, Germany). Subsequently, 15 μL of each sample were dispensed in quintuplicate onto a reusable 96-well silicon IR Biotyper^®^ plate (Bruker Daltonics GmbH, Bremen, Germany), with each spot serving as a technical replicate. For database generation, each isolate was sub-cultivated for 2 or 3 days (biological replicates) to accommodate technical variability. Additionally, 10 μL of the two standards, IRTS1 and IRTS2 (IR Biotyper^®^ kit, Bruker Daltonics GmbH, Bremen, Germany), were included in duplicate on each plate per run (Bruker, 2021; Figure 1C). One spot was left empty to measure the background after each spot. Spectra of poor quality, such as those with absorbance values outside the 0.4–2.0 window, were excluded from analysis, aiming to include at least 12–15 spectra of each isolate.

### 2.5 FT-IR spectra

Spectra were acquired (transmission mode between wave numbers 4,000–500 cm^−1^) and processed by OPUS software V.8.2.28 (Bruker Optics, Germany) on an IR Biotyper^®^ with the corresponding IR Biotyper^®^ software V4.0 (Bruker Daltonics) for data analysis. Spectral splicing was performed by default settings to the polysaccharide region of 1,300–800 cm^−1^. For spectral distance visualization, dendrograms were created using the Euclidean distance as the exploration method with the UPGMA linkage type (average). Scatter plots in both 2D and 3D were visualized using dimension reduction techniques, namely Principal Component Analysis (PCA) and Linear Discriminant Analysis (LDA). To correct for technical variance, LDA was employed as a preprocessing step, capturing 95% of the variance with a maximum of 30 principal components, with isolate ID serving as the target group for the visualization of the 2D and 3D scatter plots. The isolates were labeled according to the corresponding Quellung reaction outputs to compare the results of the two techniques.

### 2.6 Classifier creation with an artificial neural network (ANN) machine learning model to classify pneumococcal serotypes

Among the 76 *S. pneumoniae* isolates, 18 distinct serotypes were identified using Quellung serology (3, 6C, 7F, 7C, 9 N, 10A, 16F, 14, 15A, 15B, 15C, 18C, 19A, 22F, 23A, 23B, 33F, and 35B). These same isolates were tested on the IR Biotyper^®^ equipment to create a database for developing a classifier capable of distinguishing the different serotypes. All the spectra generated and stored in the newly created database were incorporated into the training data set. Additionally, two other machine-learning algorithms, a support vector machine with a radial basis function (RBF) kernel and another with a linear kernel, were evaluated. However, these algorithms failed to achieve a high classification accuracy (>95%) during development and were subsequently discontinued. Subsequently, the IR Biotyper^®^ software’s machine learning algorithms were trained to process the spectra and build the classifier using an ANN, repeating this process over multiple cycles (300 cycles), and producing a confusion matrix to evaluate the accuracy of the resulting classification model. It was determined that it was not feasible to differentiate between serotypes 15ABC, 10A/16F, and 7CF; these were amalgamated into a single group for future classification models. Ultimately, the classifiers’ accuracy was tested through an internal validation trial utilizing all the generated spectra, which were split into two sets: a training dataset and a testing/validation dataset containing 384 and 459 spectra, respectively. This process also produced a confusion matrix as an output file, which depicted the accuracy of the predicted outcomes in three colors (green, red, and yellow), confirming the reliability of the serotypes classified with our developed classifier.

## 3 Results

### 3.1 Distribution of identified serotypes

**Figure 2:**
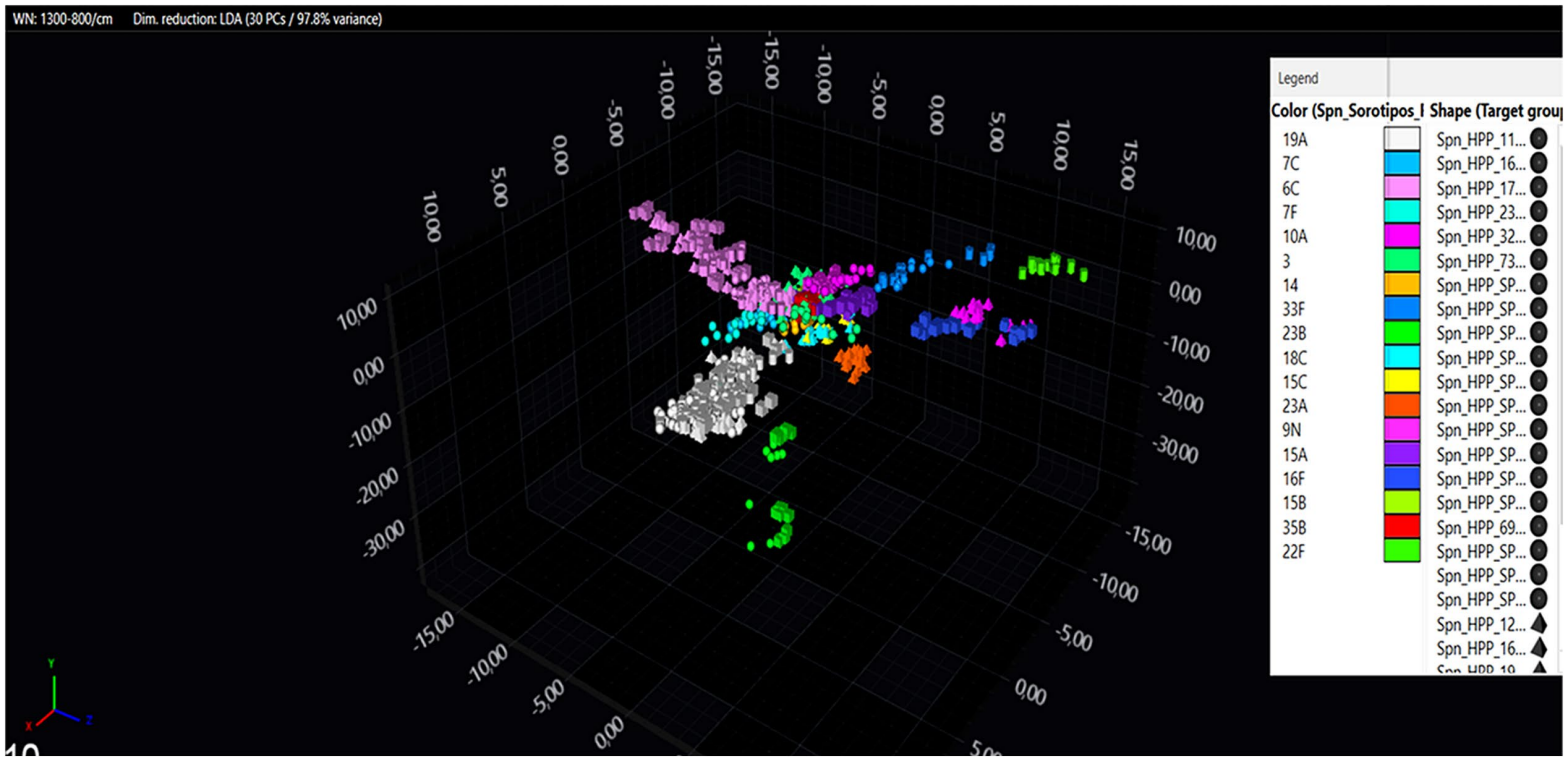
Representation of IR Biotyper results as a 3D scatter plot (LDA with 30 PCs accounting for 97.8% of the variation) from 843 spectra.

Of the 76 isolates, 48 (63.2%) were isolated from blood, 15 (19.7%) from pleural fluid, 11 (14.5%) from cerebrospinal fluid, one (1.3%) from ascitic fluid and one (1.3%) from thoracentesis fluid samples. Eighteen distinct serotypes were identified, of which serotypes 19A, 6C, and 3 were the most prevalent (Figure 2). The three most prevalent serotypes [19A (gray), 6C (light pink) and 3 (green)] show the largest “clouds” in the 3D plot (Figure 2). In this plot, the first, second and third principal components are displayed, which already show some distinct clusters. For the further analysis, all 30 principal components were explored to see distinct clusters and therefore the possibility of distinguishing/differentiating all the serotypes measured. Supplementary Figure S1 shows a deviation plot of the spectra generated by the readings of the three prevalent serotypes in our study, displaying their spectral differences. The IR Biotyper^®^ spectra for serotypes 19A, 6C, and 3 were analyzed using a deviation plot, which presents the median spectra of each serotype group along with their respective standard deviations displayed as a shade. In this deviation plot, the spectral differences between serotypes 19A, 6C, and 3 are shown, indicating that these can be differentiated using FT-IR. With the use of machine learning algorithm, which is incorporated in the IR Biotyper^®^ software, a classification model could be made. The purpose of a classifier is to evaluate spectra without the need of manual exploratory data analysis. This means that when a classification model is applied in the software (automatically triggered for pneumococci), it immediately predicts the outcome of any unknown measured spectrum such as the serotype. The created pneumococci serotype classifier can then be used in the future for automated serotype classification, during measurement of pneumococci isolates. Different serotypes can be observed in the dendrogram (Supplementary Figure S2), where the serotypes are represented in different colors on the right vertical axis. The cutoff value (8.216) of the dendrogram was an automatically calculated cutoff value (based on Simpson’s diversity index × mean coherence), which was selected to visualize the different serogroups and observe the clustering of the samples individually. The program has a limitation on the number of spectra (up to 500 spectra) to generate the dendrogram. To generate Supplementary Figure S2, the “average” function was used, which means that the spectra (both technical and biological representatives) are presented as “average spectra.” In this model, serotypes 9 N, 10A, 16F, 7C, and 7F are not distinguishable in the dendrogram, but this case does not create a problem in our classification model. For a dataset consisting of a total of 843 spectra obtained in the study, the use of a dendrogram to visualize clustering may not be recommended due to the complexity of visualizing hierarchical relationships. Dendrograms are effective for smaller datasets. An alternative to overcome this limitation is to use dimension reduction techniques, such as LDA. By applying LDA, the informative features of the spectra are retained, simplifying the data and revealing patterns that would otherwise cannot be seen in the dendrogram.

### 3.2 Classification and validation of pneumococcal serotypes

**Figure 3:**
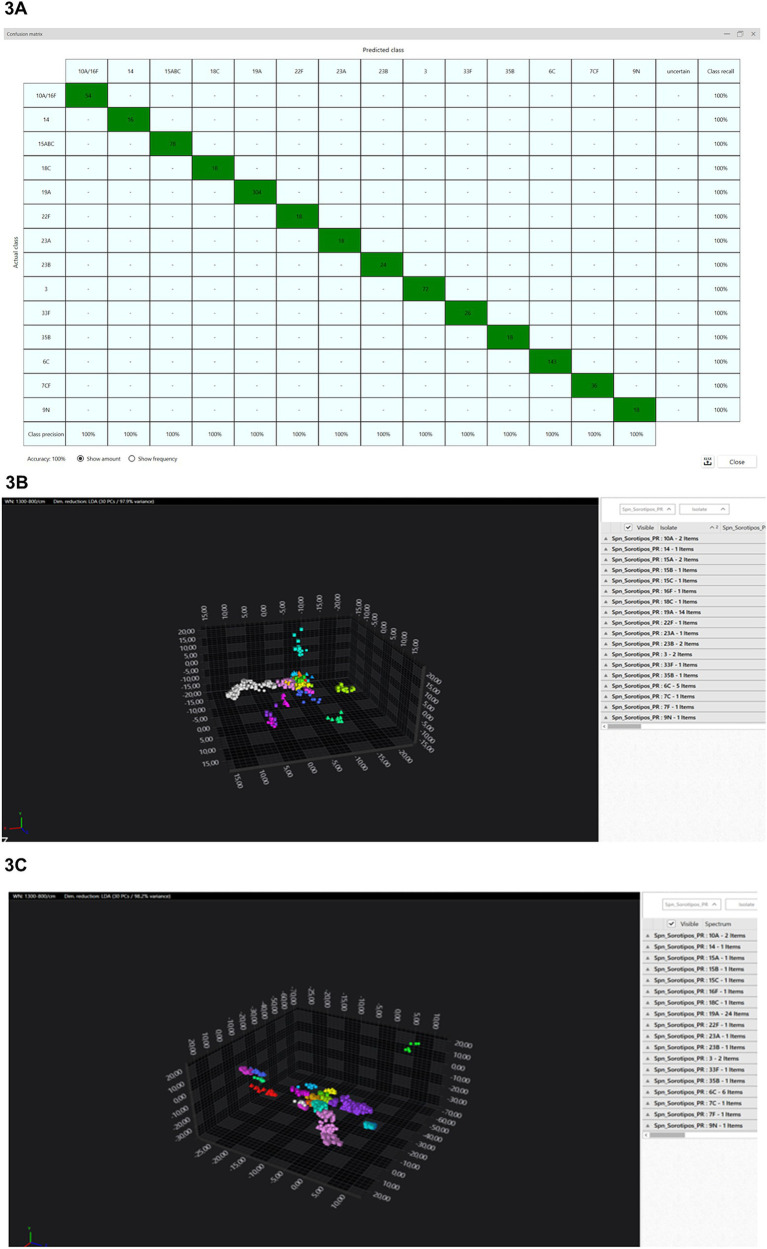
Classification and validation of pneumococcal serotypes. **(A)** Confusion matrix demonstrating the classifier’s creation from 843 spectra using ANN over 300 cycles, achieving 100% accuracy. The vertical axis displays trained labels (actual class), and the horizontal axis shows predicted classes by the classifier model. **(B)** IR Biotyper result represented as a 3D scatter plot from the classifier creation training dataset, involving 384 spectra from 39 isolates representing all 18 serotypes (LDA with 30 PCs/97.7% variance). Colors and shapes differentiate serotypes and isolates, respectively. **(C)** IR Biotyper result represented as a 3D scatter plot from the classifier creation validation dataset, involving 459 spectra from 48 isolates representing all 18 serotypes (LDA with 30 PCs/98.2% variance). Colors and shapes differentiate serotypes and isolates, respectively.

**Figure 4:**
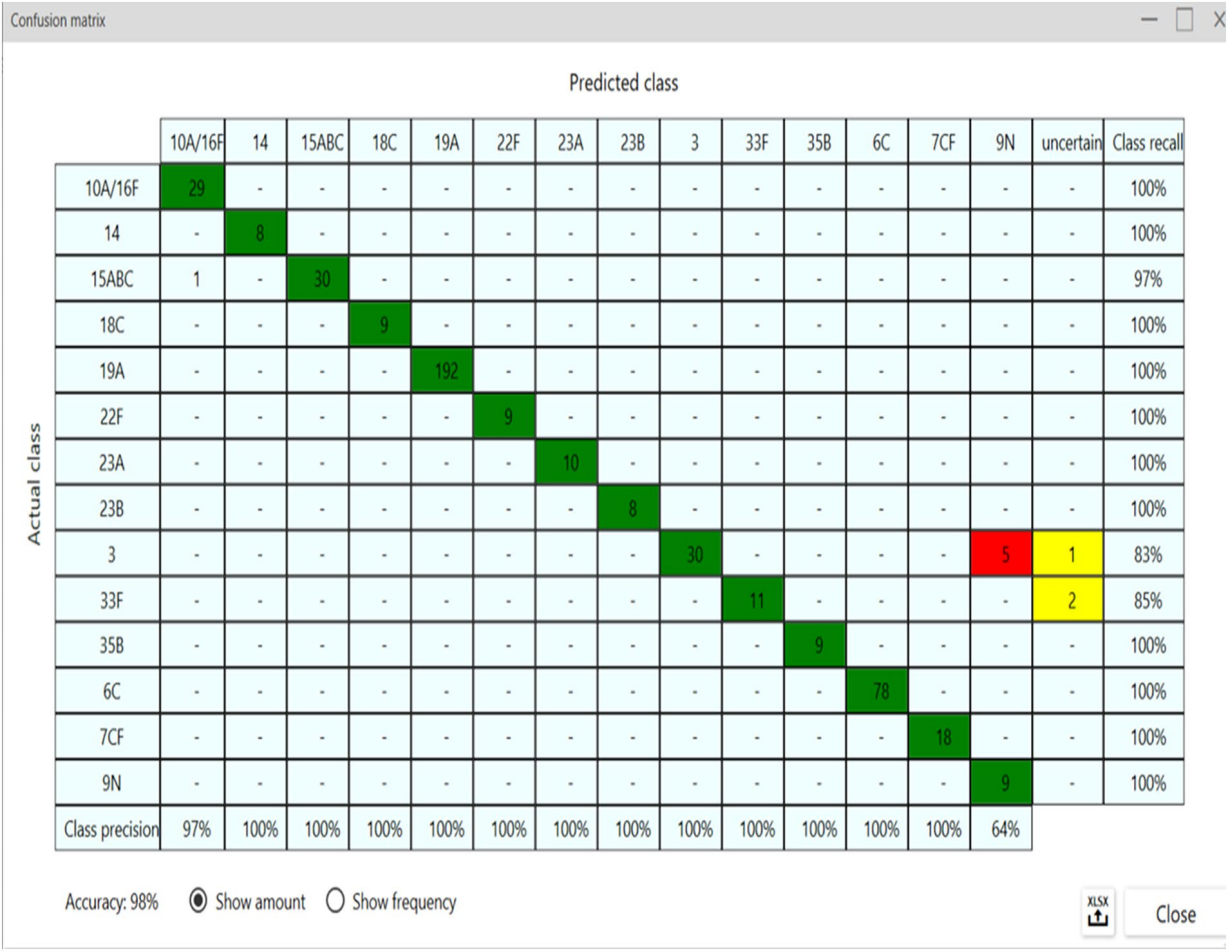
Confusion matrix for the internal validation of the classifier, showing 98% accuracy. The vertical axis displays trained labels (class recall = sensitivity), and the horizontal axis shows predicted classes (class precision = specificity), by the classifier model. Green for compatible spectra; Red for spectra crossings; Yellow for uncertain.

From the spectra obtained from 76 isolates representing 14 distinct serotypes (Table 1), a classifier was developed using all available machine learning algorithms in the IR Biotyper^®^ software. Among these algorithms, the Artificial Neural Network (ANN) machine learning demonstrated the highest possible accuracy. Upon creating the classifier, the confusion matrix immediately displayed the model’s classification accuracy. However, with the initial classifier differentiating all 14 serotypes, the accuracy threshold of >95% was not achieved due to misclassifications between serotypes 10A, 16F, 7C, 7F, 15A, 15B, and 15C, which was also visually evident in the 3D scatter plots from the LDA. For example, the confusion matrix showed that serotype 10A (actual class, vertical axis) was predicted as serotype 16F (predicted class, horizontal axis), indicating that these two serotypes could not be distinguished within this dataset. As a result, serotypes 10A and 16F were merged into a single label, 10A/16F, in the classifier. Similarly, serotypes 7C and 7F, as well as 15B and 15C, were combined. As a result, to create our final classifier, from the 76 isolates tested, 843 spectra were generated, classifying 11 distinct serotypes and three serogroups (Table 1), achieving an accuracy of 100% after 300 training cycles of the ANN (Figure 3A). The classifier was then ready for use, but before implementation, an internal validation was conducted to assess its robustness. Consequently, the entire dataset was partitioned into two groups: Group 1, the training set with “reduced” data, encompassed 384 spectra (Figure 3B); Group 2, used to test and validate the classifier, included 459 spectra (Figure 3C). Isolates in the training dataset displaying variance in their respective serotypes were selected. Following the 300-cycle training of the neural network, model using these two groups, an accuracy of 98% was achieved (Figure 4).

**Table 1:** Analysis includes *Streptococcus pneumoniae* isolates.

Serotypes	Number of isolates	Number of spectra
19A	38	304
6C	11	143
15ABC	6	78
3	4	72
10A/16F	3	54
7CF	2	36
33F	2	26
23B	3	24
18C	1	18
22F	1	18
23A	1	18
35B	1	18
9 N	1	18
14	2	16

Groupings of serotypes into their respective classification groups include 10A with 16F; 7C with 7F; and 15A, 15B, and 15C.

## 4 Discussion

IPD is of particular concern in the pediatric population because of its associated high morbidity and mortality rates. The serological types of *S. pneumoniae* demonstrate different clinical and epidemiological characteristics, and their accurate identification is crucial for the proper management of the disease and formulation of vaccine strategies (Croney et al., 2013; Thadchanamoorthy and Dayasiri, 2021). In this study, we observed that serotypes 19A, 6C, and 3 were circulating in our hospital. Serotype 19A has been reported as one of the most common serotypes isolated from IPDs and is associated with a high mortality rate, especially in the pediatric population in various medical centers (Lyu et al., 2024; Shoji et al., 2018). In Brazil, the PCV10 vaccine was introduced into the childhood National Immunization Program in 2010 and is still available for administration to children up to 1 year of age in the primary scheme of two doses (2 and 4 months) plus a booster (12 months) to complete the scheme (Brasil, Ministério da Saúde, 2023). However, the PCV10 vaccine does not cover serotypes 19A, 6C, and 3, which may explain the prevalence of isolates belonging to these serotypes recovered in our series. Data related to PCV10 vaccination coverage in the state of Paraná in 2023 were approximately 92,752% in the primary scheme doses and 82, 77% in the complete scheme with a booster dose. In 2023, the pneumonia and meningitis surveillance bulletin reported 345 serotype 19A isolates in Brazil, with 44.6% (*n* = 154/345) identified in patients aged <5 years (SIREVA, 2023). Notably, serotype 3 remains highly prevalent, even in countries that have incorporated high-valence PCVs, including serotype 3 in their formulation (PCV13, PCV15, and PCV20) (Bertran et al., 2024). This prevalence is attributed to the capsule synthesis pathway, where modifications in glycosidic bonds lead to thicker capsules, forming mucoid colonies. Furthermore, *S. pneumoniae* serotype 3 produces and releases capsules during cell growth, which inhibits antibody opsonization. Clinically, this can result in vaccination failures, resistance to phagocytosis, and subsequent respiratory infections (Choi et al., 2016; Paton and Trappetti, 2019; Yang et al., 2021). The Quellung reaction, performed at the reference laboratory IAL in Brazil, remains the gold standard for serological typing of *S. pneumoniae* strains, despite its limitations such as the variety, quantity, and cost of antisera required, typically restricted to reference centers with the necessary expertise. Conversely, the FT-IR serotyping method offers a promising alternative, significantly reducing the time to obtain results compared to traditional methods and at a lower cost. The IR Biotyper^®^ software facilitates immediate, real-time classification of 34 distinct serotypes of *S. pneumoniae* using the Bruker Classifier included with the IR Biotyper^®^ 4.0 software However, it is important to note that this classification model was developed by manufacturers using isolates grown on Columbia blood agar with a smear protocol after UV inactivation (Passaris et al., 2022). In this study, we were use IR Biotyper^®^ suspension vials for sample preparations. In the context of this project, a custom classifier was developed specifically to predict serotypes in real-time in local circulating serotype analysis of pneumococcal samples in Paraná, Brazil. This classification model, with ANN machine learning integrated into the IR Biotyper^®^ software, achieved an accuracy is 98%, based on the training dataset. Furthermore, the IR Biotyper^®^ software allows users to retrain the classifier by modifying its training dataset, providing flexibility to update and improve its predictive model, thus increasing its robustness. In summary, the IR Biotyper^®^ is proving to be a promising tool for the phenotypic classification of *S. pneumoniae* serotypes. Validation of this technology will make it possible to monitor circulating serotypes in cases of IPD with a quick turnaround time and low cost, with a positive impact on clinical and epidemiological aspects, vaccine production, and patient management. In addition, it allows for the expansion of this technology to other medical and research centers that are not restricted to reference laboratories. One limitation is that, for some of the serotypes we found, we only had one isolate. According to the IR Biotyper^®^ software manual, it is recommended to have several spectra per isolate from independent cultivations. For this reason, we increased the number of spectra, including technical and biological variations, for classifier training and internal validation to classify them accurately. In the future, it is necessary to include more of these less prevalent isolates to re-train the classifier model and increase the sensitivity and specificity of the model for these serotypes. However, this did not affect our study because these were not the most prevalent serotypes in our setting. For the bacterial suspension protocol proposed by the manufacturer, a high biomass is required in this suspension, which can be a challenge for *S. pneumoniae*. It is also important to note that this classification model should be tested with an external validation set, which is a recommended step before implementing it in routine diagnostic use. Considering that this is a phenotypic test, it is important to consider and include precautions that must be taken to obtain reproducible results, such as following the manufacturer’s culture medium respecting the same composition and the recommended incubation temperature.

## Data Availability

The original contributions presented in the study are included in the article/Supplementary material, further inquiries can be directed to the corresponding author.

## References

[ref1] Abdul Rahman N. A. Mohd Desa M. N. Masri S. N. Taib N. M. Sulaiman N. Hazman H.. (2023). The molecular approaches and challenges of *Streptococcus pneumoniae* serotyping for epidemiological surveillance in the vaccine era. Polish J. Microbiol. 72, 103–115. doi: 10.33073/pjm-2023-023, PMID: 37314355 PMC10266295

[ref2] ANVISA AN de VS. Prevenar^®^ 20 (Vacina pneumocócica 20-valente conjugada): novo registro [Internet]. (2023). Available at: https://www.gov.br/anvisa/pt-br/assuntos/medicamentos/novos-medicamentos-e-indicacoes/prevenar-r-20-vacina-pneumococica-20-valente-conjugada-novo-registro

[ref3] Bardach A. Ruvinsky S. Palermo M. C. Alconada T. Sandoval M. M. Brizuela M. E.. (2024). Invasive pneumococcal disease in Latin America and the Caribbean: serotype distribution, disease burden, and impact of vaccination. A systematic review and meta-analysis. PLoS One 19. doi: 10.1371/journal.pone.0304978 PMC11210815 38935748

[ref4] Bertran M. D’Aeth J. C. Abdullahi F. Eletu S. Andrews N. J. Ramsay M. E.. (2024). Invasive pneumococcal disease 3 years after introduction of a reduced 1 + 1 infant 13-valent pneumococcal conjugate vaccine immunisation schedule in England: a prospective national observational surveillance study. Lancet Infect. Dis. 24, 546–556. doi: 10.1016/S1473-3099(23)00706-5, PMID: 38310905

[ref5] Brasil, Ministério da Saúde (2023). Calendário Nacional de Vacinação da Criança. Brasil:Ministério da Saúde. Available at: https://www.gov.br/saude/pt-br/vacinacao/calendario (Accessed November 13, 2024).

[ref6] Bruker. IR Biotyper user manual. (2021) REF 1845471, May 2022. Doc. no. 5025119 Revision F.

[ref7] Burckhardt I. Sebastian K. Mauder N. Kostrzewa M. Burckhardt F. Zimmermann S. (2019). Analysis of *Streptococcus pneumoniae* using Fourier-transformed infrared spectroscopy allows prediction of capsular serotype. Eur. J. Clin. Microbiol. Infect. Dis. 38, 1883–1890. doi: 10.1007/s10096-019-03622-y, PMID: 31286288 PMC6778537

[ref8] Carvalho M. D. G. Pimenta F. C. Jackson D. Roundtree A. Ahmad Y. Millar E. V.. (2010). Revisiting pneumococcal carriage by use of broth enrichment and PCR techniques for enhanced detection of carriage and serotypes. J. Clin. Microbiol. 48, 1611–1618. doi: 10.1128/JCM.02243-09, PMID: 20220175 PMC2863911

[ref9] Choi E. H. Zhang F. Lu Y. Malley R. (2016). Capsular polysaccharide (CPS) release by serotype 3 pneumococcal strains reduces the protective effect of anti-type 3 CPS antibodies 23, 162–167. doi: 10.1128/CVI.00591-15 PMC4744920 26677201

[ref10] Croney C. M. Nahm M. H. Juhn S. K. Briles D. E. Crain M. J. (2013). Invasive and noninvasive *Streptococcus pneumoniae* capsule and surface protein diversity following the use of a conjugate vaccine. Clin. Vaccine Immunol. 20, 1711–1718. doi: 10.1128/CVI.00381-13, PMID: 24006139 PMC3837785

[ref11] Donkor E. S. (2013). Molecular typing of the pneumococcus and its application in epidemiology in sub-Saharan Africa. Front. Cell. Infect. Microbiol. 3:12. doi: 10.3389/fcimb.2013.00012 23503978 PMC3596783

[ref12] Ganaie F. Maruhn K. Li C. Porambo R. J. Elverdal P. L. Abeygunwardana C.. (2021). Structural, genetic, and serological elucidation of *Streptococcus pneumoniae* serogroup 24 serotypes: discovery of a new serotype, 24C, with a variable capsule structure. J. Clin. Microbiol. 59:e0054021. doi: 10.1128/JCM.00540-21, PMID: 33883183 PMC8218768

[ref13] Ganaie F. A. Saad J. S. Lo S. W. McGee L. Bentley S. D. van Tonder A. J.. (2023a). Discovery and characterization of pneumococcal serogroup 36 capsule subtypes, serotypes 36A and 36B. J. Clin. Microbiol. 61:e0002423. doi: 10.1128/jcm.00024-23, PMID: 36971549 PMC10117043

[ref14] Ganaie F. A. Saad J. S. Lo S. W. McGee L. van Tonder A. J. Hawkins P. A.. (2023b). Novel pneumococcal capsule type 33E results from the inactivation of glycosyltransferase WciE in vaccine type 33F. J. Biol. Chem. 299:105085. doi: 10.1016/j.jbc.2023.105085, PMID: 37495106 PMC10462825

[ref15] Ganaie F. Saad J. S. Mcgee L. Van T. A. J. Bentley S. D. Lo S. W.. (2020). A new pneumococcal capsule type, 10D, is the 100th serotype and has a large cps fragment from an oral *Streptococcus* 11:e00937-20. doi: 10.1128/mBio.00937-20 PMC7240158 32430472

[ref16] Habib M. Porter B. D. Satzke C. (2014). Capsular serotyping of *Streptococcus pneumoniae* using the quellung reaction. J. Vis. Exp. 84, 1–4. doi: 10.3791/51208 PMC4131683 24637727

[ref17] Jarovsky D. Berezin E. N. (2023). Impact of PCV10 on pediatric pneumococcal disease burden in Brazil: time for new recommendations? J. Pediatr. 99, S46–S56. doi: 10.1016/j.jped.2022.11.003, PMID: 36495946 PMC10066423

[ref18] Kfouri R. A. Brandileone M. Cristina C. Richtmann R. Gilio A. E. S. P. (2023). Chronic medical conditions associated with invasive pneumococcal diseases in inpatients in teaching ~ o Paulo city: estimating antimicrobial hospitals in S a susceptibility and serotype-coverage of pneumococcal vaccines 27, 102746. doi: 10.1016/j.bjid.2023.102746, PMID: 36758625 PMC9943857

[ref19] Lages P. M. Carlesse F. Boettger B. C. Pignatari A. C. C. Petrilli A. S. de Moraes-Pinto M. I. (2020). Invasive pneumococcal disease in children with cancer: incidence density, risk factors and isolated serotypes. Braz. J. Infect. Dis. 24, 489–496. doi: 10.1016/j.bjid.2020.09.003, PMID: 33164827 PMC9392108

[ref20] Lyu S. Shi W. Dong F. Xu B. P. Liu G. Wang Q.. (2024). Serotype distribution and antimicrobial resistance of pediatric *Streptococcus pneumoniae* isolated from inpatients and outpatients at Beijing Children’s hospital. Braz. J. Infect. Dis. 28:103734. doi: 10.1016/j.bjid.2024.103734, PMID: 38471654 PMC11004498

[ref21] Manna S. Werren J. P. Ortika B. D. Bellich B. Pell C. L. Nikolaou E.. (2024). *Streptococcus pneumoniae* serotype 33G: genetic, serological, and structural analysis of a new capsule type. Microbiol. Spectr. 12:e0357923. doi: 10.1128/spectrum.03579-23 38059623 PMC10782959

[ref22] Muchaamba F. Stephan R. (2024). A comprehensive methodology for microbial strain typing using Fourier-transform infrared spectroscopy. Methods Protoc. 7. doi: 10.3390/mps7030048, PMID: 38921827 PMC11207048

[ref23] Novais Â. Freitas A. R. Rodrigues C. Peixe L. (2019). Fourier transform infrared spectroscopy: unlocking fundamentals and prospects for bacterial strain typing. Eur. J. Clin. Microbiol. Infect. Dis. 38, 427–448. doi: 10.1007/s10096-018-3431-3 30483997

[ref24] Passaris I. Mauder N. Kostrzewa M. Burckhardt I. (2022). Validation of Fourier transform infrared spectroscopy for serotyping of *Streptococcus pneumoniae*. J. Clin. Microbiol. 60:e0032522. doi: 10.1128/jcm.00325-22, PMID: 35699436 PMC9297836

[ref25] Paton J. C. Trappetti C. (2019). *Streptococcus pneumoniae* capsular polysaccharide. Gram Posit. Pathog., 304–315. doi: 10.1128/9781683670131.ch19 PMC11590643 30977464

[ref26] Rappuoli R. De G. E. Costantino P. Smith D. Anderson P. Anderson P.. (2019). On the mechanisms of conjugate vaccines. PNAS 116, 14–16. doi: 10.1073/pnas.1819612116 30578318 PMC6320500

[ref27] Qian D. Q. Shi W. Yu D. Hu Y. K. (2021). Epidemiology of non-vaccine serotypes of *Streptococcus pneumoniae* before and after universal administration of pneumococcal conjugate vaccines. Hum. Vaccin. Immunother. 17, 5628–5637. doi: 10.1080/21645515.2021.1985353, PMID: 34726580 PMC8903918

[ref28] Shoji H. Vázquez-Sánchez D. A. Gonzalez-Diaz A. Cubero M. Tubau F. Santos S.. (2018). Overview of pneumococcal serotypes and genotypes causing diseases in patients with chronic obstructive pulmonary disease in a Spanish hospital between 2013 and 2016. Infect. Drug Resist. 11, 1387–1400. doi: 10.2147/IDR.S165093, PMID: 30214260 PMC6128270

[ref29] Silva-Costa C. Brito M. J. Pinho M. D. Friães A. Aguiar S. I. Ramirez M.. (2018). Pediatric complicated pneumonia caused by *streptococcus pneumoniae* serotype 3 in 13-valent pneumococcal conjugate vaccinees, Portugal, 2010–2015. Emerg. Infect. Dis. 24, 1307–1314. doi: 10.3201/eid2407.180029, PMID: 29912700 PMC6038763

[ref30] SIREVA (2023). Secretaria de Estado da Saúde. Coordenadoria de Controle de Doenças Instituto Adolfo Lutz. Informação da vigilância das pneumonias e meningites bacterianas: Instituto Adolfo Lutz. Available at: http://www.ial.sp.gov.br/resources/insituto-adolfo-lutz/publicacoes/sireva_2023_2.pdf (Accessed November 13, 2024).

[ref31] Swarthout T. D. Gori A. Bar-Zeev N. Kamng’ona A. W. Mwalukomo T. S. Bonomali F.. (2021). Evaluation of pneumococcal serotyping of nasopharyngeal-carriage isolates by latex agglutination, whole-genome sequencing (PneumoCaT), and DNA microarray in a high-pneumococcal-carriage-prevalence population in Malawi. J. Clin. Microbiol. 59, 59:e02103-20. doi: 10.1128/JCM.02103-20 PMC7771446 33087431

[ref32] Thadchanamoorthy V. Dayasiri K. (2021). Review on pneumococcal infection in children. Cureus 13, 1–7. doi: 10.7759/cureus.14913 PMC8189266 34123613

[ref33] Velusamy S. Tran T. Mongkolrattanothai T. Walker H. McGee L. Beall B. (2020). Expanded sequential quadriplex real-time polymerase chain reaction (PCR) for identifying pneumococcal serotypes, penicillin susceptibility, and resistance markers. Diagn. Microbiol. Infect. Dis. 97:115037. doi: 10.1016/j.diagmicrobio.2020.115037, PMID: 32265073

[ref34] Weiser J. N. Ferreira D. M. Paton J. C. (2018). *Streptococcus pneumoniae*: transmission, colonization and invasion. Nat. Rev. Microbiol. 16, 355–367. doi: 10.1038/s41579-018-0001-8, PMID: 29599457 PMC5949087

[ref35] WHO (2011). *Neisseria meningitidis*, *Streptococcus pneumoniae*, diagnosis of meningitis caused by laboratory methods for the and *Haemophilus influenzae*: WHO manual. 2nd Edn: World Health Organizarion.

[ref36] Yang Y. Zhen H. C. Fang C. Ping X. Y. Li W. Fu Y. (2021). Properties of mucoid serotype 3 *Streptococcus pneumoniae* from children in China 11, 1–8. doi: 10.3389/fcimb.2021.648040 PMC8024565 33842394

