# Supplementary figures and images for: Fourier transform infrared spectroscopy for Streptococcus pneumoniae capsular serotype classification in pediatric patients with invasive infections
Source: Front Microbiol. 2024 Nov 21;15:1497377. doi: 10.3389/fmicb.2024.1497377 (PMC11619633; doi:10.3389/fmicb.2024.1497377)

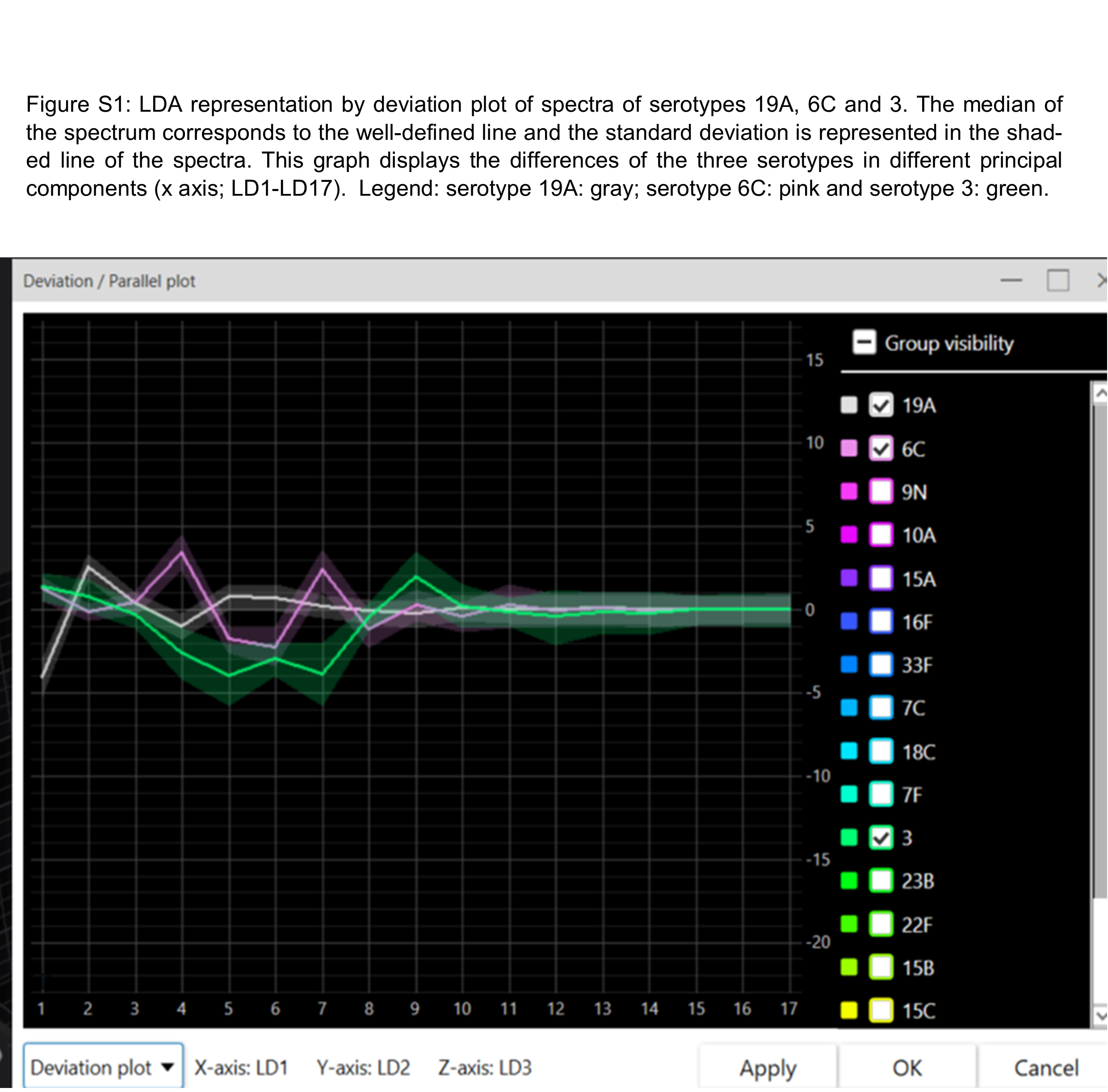

Supplement: Supplementary file 1 [file Image_1.tif]

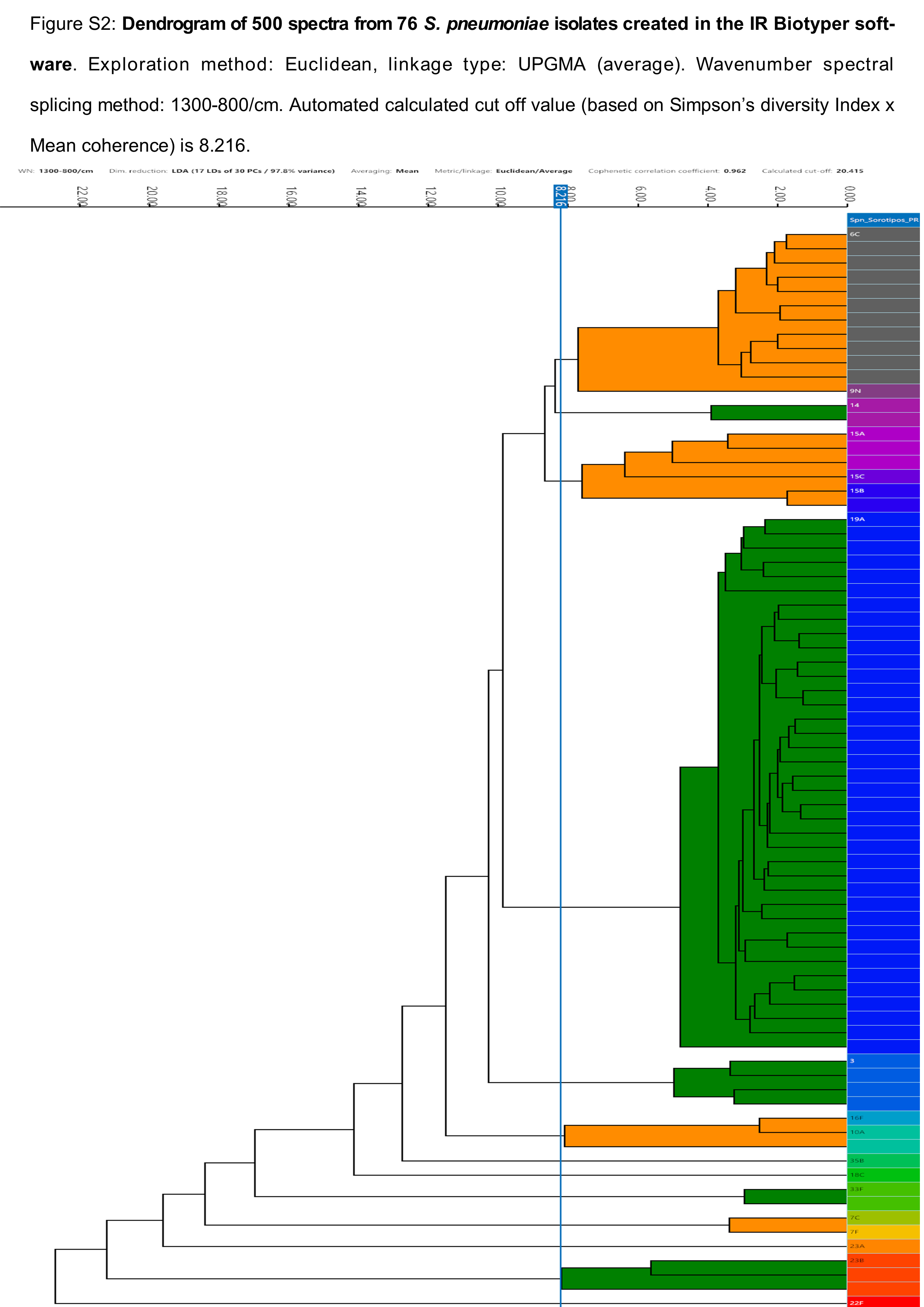

Supplement: Supplementary file 2 [file Image_2.tif]
